# Comparison between breath stacking technique associated with expiratory muscle training and breath stacking technique in amyotrophic lateral sclerosis patients: Protocol for randomized single blind trial

**DOI:** 10.1016/j.conctc.2020.100647

**Published:** 2020-09-02

**Authors:** Alessandra Dorça, Livia A. Alcântara, Denise Sisterolli Diniz, Max Sarmet, Sérgio Ricardo Menezes Mateus, Luis Vicente Franco Oliveira, Hamilton Franco, Vinicius Maldaner

**Affiliations:** a Department of Speech Sciences, Federal University of Goias, Goias, Brazil; b Reference Center of Neuromuscular Diseases, Hospital de Apoio de Brasília, Brasília, Brazil; c Physical Therapy Department, University of Brasilia, Brasília, Brazil; d Post Graduate ProgramHuman Movement and Rehabilitation Program, University Center of Anapolis UniEvangelica, Anapolis, Brazil

**Keywords:** Amyotrophic lateral sclerosis, Assisted coughing, Breathing exercises

## Abstract

**Introduction:**

Amyotrophic lateral sclerosis (ALS) is a progressive neurodegenerative disease affecting both upper and lower motor neurons, and lead to respiratory failure. Strategies are suggested to respiratory management in ALS patients, as the breath stacking and Expiratory muscle training (EMT), which have been used as aid to assist cough in neuromuscular disorders. However, the randomized controlled trials performed in ALS patients have not investigated the addiction of EMT together breath stacking in this population. This trial aims to determine if breath stacking plus EMT is more effective than breath stacking alone to decrease the decline rate on the inspiratory/expiratory muscle strength, FVC and voluntary PCF in ALS patients.

**Methods:**

This parallel-group, assessor-blinded randomized controlled trial, powered for superiority, aims to assess pulmonary function, respiratory muscle strength, peak cough flow as primary outcomes. Forty-two participants are being recruited referral neuromuscular disease center at Brasilia, Brazil. Following baseline testing, participants are randomized using concealed allocation, to receive either: a) breath stacking technique alone or b) breath stacking technique plus EMT.

**Conclusion:**

There is a lack of evidence regarding the benefit of EMT plus breath stacking in ALS patients. This trial will contribute to evidence currently being generated in national and international trials by implementing and evaluating a respiratory therapy program including two components not yet combined in previous research, for people with ALS involving longer-term follow-up of outcomes. This trial is ongoing and currently recruiting.

**Trial registration:**

This trial was prospectively registered on the Clinical Trials Registry NCT04226144.

## Highlights

• Amyotrophic lateral sclerosis (ALS) is a progressive neurodegenerative disease that leading respiratory impairment.
• Strategies to enhance effective cough and lung recruitment should be recommended in ALS patients.
• Breath stacking and expiratory muscle training (EMT) are tools that improve airway clearance. the randomized controlled trials performed in ALS patients have not investigated the addiction of EMT together breath stacking in this population.
• We believe that those receiving breath stacking plus EMT will have a slower decline in these parameters from baseline to 6 months, compared to patients that receiving breath stacking alone.

## 1 Introduction

Amyotrophic lateral sclerosis (ALS) is a progressive neurodegenerative disease affecting both upper and lower motor neurons, which leads to a loss of voluntary muscle control [1]. As the disease progresses, muscles of respiration are affected, leading to chronic respiratory failure [2]. Respiratory failure is primarily due to respiratory muscle weakness, leading ALS patients to cough impairment, one of the main causes of hospitalization [3,4]. Producing an effective cough initially requires taking a deep breath with the inspiratory muscles, followed by maximum contraction of the expiratory muscles, with glottis closure and subsequent opening using the oropharyngeal muscles, generating an expiratory flow due to contraction of expiratory muscles capable of eliminating secretions [5,6]. This represents the inspiratory, compression and expiratory phases of cough production. Thus, strategies to enhance effective cough and lung recruitment should be recommended in ALS patients [7].

Among these strategies suggested for respiratory management in ALS patients, breath stacking has been used to assist cough in neuromuscular disorders [8]. The breath-stacking technique uses a manual resuscitation bag with one-way valve to deliver large breath volumes to the patient via a suitable interface [9]. Periodic lung expansion by breath stacking decreases basal atelectasis, and maintains compliance of the lungs and chest wall, with increases of Peak cough flow (PCF) and cough effectiveness [5]. Rafiq et al. have demonstrated that breath stacking should be recommended as a first line intervention for lung recruitment and cough augmentation in ALS patients, with no differences between breath stacking and mechanical insufflator-exsufflator for survival, quality of life and hospitalization [9].

Expiratory muscle training (EMT) has been investigated as a new effective tool to enhance maximal PCF in individuals with neuromuscular diseases. This technique aims to improve subglottic air pressure generation, swallowing and airway clearance [10]. Reyes et al. have shown that EMT plus breath stacking improved voluntary and reflex cough in Parkinson disease [11]. Plowman et al. have demonstrated that 5-week EMT training was feasible and led to improvements in respiratory and bulbar function in ALS patients [10,12]. However, both studies have not used the techniques together, which may improve the clinical results. Of note, the randomized controlled trials performed in ALS patients have not investigated the addiction of EMT together breath stacking in this population.

This trial aims to determine if breath stacking plus EMT is more effective than breath stacking alone to decrease the decline rate on the inspiratory/expiratory muscle strength, pulmonary function testing and voluntary PCF in ALS patients. We hypothesize that those receiving breath stacking plus EMT will have a slower decline in these parameters from baseline to 6 months, compared to patients that receiving breath stacking alone. The key secondary outcomes are to assess whether EMT plus breath stacking is superior to breath stacking alone to swallowing function and ability to speech. This protocol is reported according to the Standard Protocol Items: Recommendations for interventional Trials (SPIRIT guidance) [13].

### 1.1 Methods/design

This two-arm, parallel (1:1), superiority, blinded-assessor, randomized controlled trial is being conducted at referral neuromuscular disease center in Brasilia, Brazil. This trial has been prospectively registered on Clinical Trials Registry (NCT04226144). Recruitment commenced in August 2019.

### 1.2 Participants

**Fig. 1:**
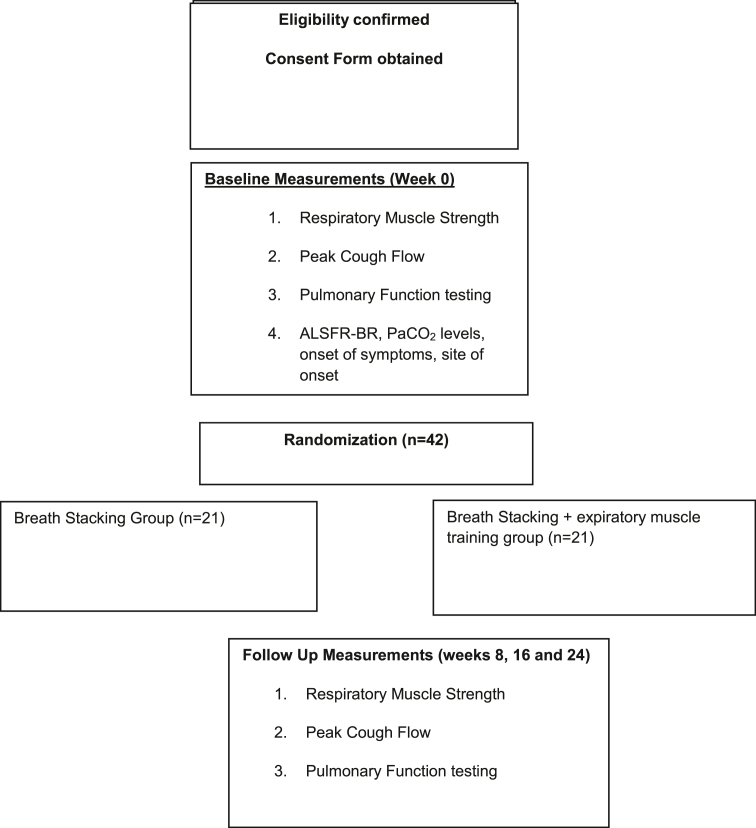
VUP valve used in Expiratory Muscle Training Program.

Fig. 1 outlines participants’ flow through the study. Eligible participants are identified through screening at the neuromuscular disease center. To be eligible, participants must have a diagnosis of neuromuscular disease confirmed by neurologists at the referral center for neuromuscular diseases in Brasília prior to screening for recruitment. Other eligibility requirements are: age over 18 years; preserved cognition, as evidenced by a score greater than or equal to 24 points in the Mini-Mental Status Exam; no barium allergies; without tracheostomy or invasive mechanical ventilation; no diaphragmatic pacemaker; and no associated respiratory disease. Participants are excluded if they are pregnant; had previous kidney disease or other concomitant diseases; had respiratory diseases; or are hospitalized in intensive care units (ICUs) during the study.

Eligible participants are contacted by the trial coordinator who explains the study aims and requirements, and those expressing interest in the study are provided with a Patient Information and Consent Form. All participants provide written informed consent prior to completing baseline outcome measures. Recruited participants may choose to withdraw from the study at any stage. Data collected prior to the time of study withdrawal will be included in data analyses.

### 1.3 Randomization and allocation

Following informed consent and all baseline assessments, participants will be randomized 1:1 to either the intervention group (EMT plus breath stacking) or the control group (breath stacking alone-usual care). A stratified block permutation randomization will be used with bulbar and non-bulbar onset symptoms as the stratification factors, to ensure balance between the groups. The patients will be dichotomized into normal to moderate bulbar impairment (score 7–12) and severe bulbar impairment (score 0–6).

The randomization schedule was prepared by a researcher independent of the trial.

The trial is being conducted in accordance with theDeclaration of Helsinki and was approved by the Fundação de Ensino e Pesquisa em Ciências da Saúde (FEPECS) Ethics Committee and received approval 6/18/2019 (Document number 3400412).

### 1.4 Interventions

#### 1.4.1 Control group

This group will just perform the breath stacking technique. For this, the patients will have a choice between using an inflated oronasal mask (Lumiar Health Care, Sao Paulo, Brazil) or a mouthpiece (Respironics, Utah, USA). The caregivers of the patients will be trained by the research-coordinators (VM, AD and SM) to perform the technique at least two sessions per day, one in the morning and one at bedtime. The caregivers will be monitored continuedly, with monthly visits to the referral center to ensure that the technique is performed well. All research coordinators have experience with the techniques and worked with respiratory management in ALS patients for at least 3 years.

To performed the breath stacking technique, we utilize the protocol previously described by Bach et al. [5]. The lungs are inflated as fully as possible by stacking successive breaths without expiration until the patients’ lung insufflation capacity (LIC). The participant will be instructed to sustain the air in the lung, closing the glottis. Once the lungs are maximally inflated, the compressed air volume is released under expiratory muscle force, thus generating a cough with lung and chest wall recoil. They will perform 5–8 cycles of breath stacking per session, stacking 3–5 breaths per cycle.

#### 1.4.2 Experimental group

**Fig. 2:**
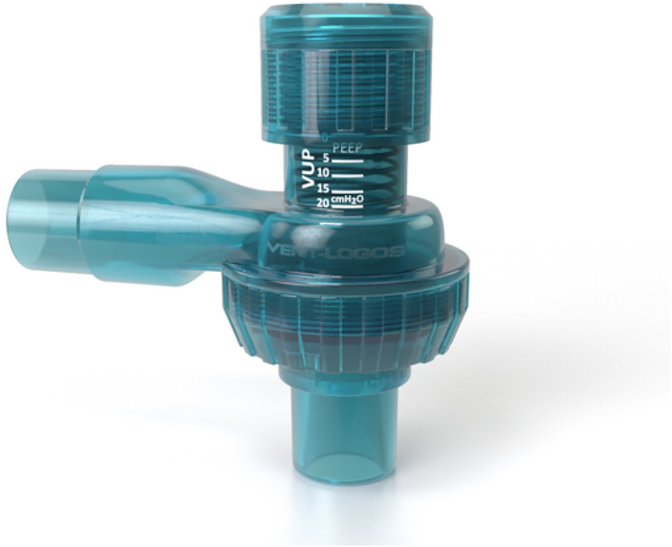
VUP valve used in EMT program.

This group will perform the breath stacking technique in addiction with EMT. The valve will be changed to the one-way valve called VUP (Lumiar, Sao Paulo, Brazil), a one-way valve (Fig. 2) that allows patients blow out air with a counter resistance during all expiratory phase.

. The initial expiratory pressure will be 8 cmH_2_O and can be changed at each visit depending on participants’ tolerance, whether it is easy or difficult to exhale assessed by research coordinators. The participants will be encouraged to blow out as slowly that they can. Both protocols will be performed for 24 weeks, and outcome measurements will be performed every 8 weeks.

#### 1.4.3 Safety and adverse events reporting

A serious adverse event is defined in this study as any event occurring either during or up to 60 min following the trial intervention or outcome assessment that is life threatening or results in death, hospitalization or prolongation of existing hospitalization, disability or incapacity. Minor adverse events directly relating to intervention or outcome measure sessions can include: aerophagia, severe breathlessness, new or progressive pain, altered mental status, palpitations and progressive fatigue [4]. Following each intervention and outcome measure session trial staff are required to complete data entry forms indicating if a serious or minor adverse event has occurred. In the case of serious adverse events the study chief investigator is notified immediately, participants are managed appropriately, and the incident will be reported to the relevant hospital ethics committee.

#### 1.4.4 Outcomes

Outcomes will be assessed during individual appointments at baseline, 8 weeks, 16 weeks and 24 weeks by a research assistant blinded to group allocation. At baseline, demographic and clinical details will be recorded, including age, sex, location of onset of symptoms, time of diagnosis, treatment details and social history. Survival data will be collected after 2 years post study recruitment.

Treatment efficacy will be determined by changes in decline rate of the primary outcomes (Maximal Inspiratory Pressure [MIP], Maximal Expiratory Pressure [MEP], Peak Cough Flow [PCF], Forced Vital Capacity [FVC) and Slow Vital Capacity [SVC]) from baseline to 24 weeks.

##### 1.4.4.1 Pulmonary function and respiratory muscle strength

The PFTs will be measured using a Vitalograph 6800 PFT spirometer (Vitalograph Inc, USA) and the decision whether to use the mouthpiece with nasal clip or the mask will be based on the evaluation of each single patient, specifically on the presence of facial muscle weakness. The PFTs that will be included are the FVC and SVC, which will be performed in both and supine positions; they are expressed both as absolute value, in FVC% and SVC%, respectively) [14]. The patient is guided to inhale deeply and, subsequently, to expire as fast as far they could.

Maximal inspiratory (MIP), and maximal expiratory pressure (MEP) were measured as previously described by Black & Hyatt [15]. Sniff nasal inspiratory pressure (SNIP) will be measured at functional residual capacity as previously described [16]. The highest value from three or more attempts will be selected for analyses and standardized to predicted percentages of Brazilian population [17].

##### 1.4.4.2 Peak cough flow (PCF) and assisted PCF

The maneuver, expressed in L/min, will be performed with a mask of fitting size, with a seated patient who is asked to inhale as much air as possible and then cough it out. At least three acceptable and repeatable trials were performed, and the best out of these maneuvers will be chosen.

PCF and manually assisted PCF by LIC will be measured using a sealed oronasal mask with a handheld portable peak flow meter (Respironics INC. USA), which recent guidelines recommend for measuring expiratory flow rates during voluntary cough [18].

##### 1.4.4.3 Bulbar function

Bulbar function will be assessed using the three components of the revised ALS functional rating scale (ALSFRS-R) relevant to bulbar function (i.e. speech, salivation and swallowing, each scored on a scale of 0–4).

##### 1.4.4.4 Secondary outcomes

Data on blood gases will be collected to assess PaCO_2_ levels at baseline. The Amyotrophic Lateral Sclerosis Functional Rating Scale revised score (ALSFR-r), site of onset [bulbar(B) versus non-bulbar (NB)], time elapsed (months) between symptom onset and ALS diagnosis, months elapsed from diagnosis to noninvasive ventilation (NIV) prescription will also be collected. Voice parameters will be assessed by Maximum Phonation Time and its acoustic analysis.

The Functional Oral Intake Scale (FOIS) [19] will be used as an index of daily oral intake. This validated 7-point ordinal scale measures what foods an individual consumes to meet their daily nutritional and hydration requirements and ranges from a 1 (nothing by mouth) to 7 (full oral diet with no restrictions). The Eating Assessment Tool-10 (EAT-10)was administered as a patient reported outcome (PRO) of swallowing function [20]. The EAT-10 is a validated 10-item patient rated questionnaire with each question rated on a 5-point ordinal scale. A total EAT-10 score ranges from 0 (indicative of no self-perceived swallowing impairments) to 40 (indicative of severe swallowing impairments).

### 1.5 Sample size calculation

This study is powered to detect a clinical meaningful difference in decline rate of pulmonary function (% predicted FVC). Based on meta-analysis conducted by Silva et al. [21], the minimal clinical important difference (MCID) from baseline to 1 year is 2–6%, 16 participants for each arm will need to be recruited with 80% power at two-tailed 5% level of significance. After take into account 30% attrition, a total sample size of 42 participants is required.

### 1.6 Statistical analysis

Baseline characteristics will be summarized, including age, sex, time of diagnosis, onset of symptoms local, drugs, primary and secondary outcomes, and imbalances will be investigated.

For each variable, the Shapiro Wilk test will be used to evaluate the normality of the distribution and the Levene test will be performed to evaluate the homogeneity of variance. The differences between groups will be analyzed by the unpaired *t*-test and for continuous variables and chi-square test for categorical variables. The decline rate will be calculated as the difference between baseline and final observation, dividing the result by the number of months between baseline and last evaluation. Spearman test will be used to evaluate the longitudinal decline correlations between pulmonary function tests and both bulbar impairment and disease progression rate.

Exploratory analysis of 2-year survival will include descriptive Kaplan-Mayer survival curves and cox regression with treatment intent.

## 2 Discussion

This study will assess the impact of expiratory muscle training associated with breath stacking technique on pulmonary function, respiratory muscle strength, peak cough flow and functional assessment in ALS patients. We chose the pulmonary function parameters and cough efficacy as primary outcomes because they plays a critical role in predicting the prognosis of these patients, both in terms of survival and functional ability [22].

A recent study suggests that expiratory muscle training was feasible and well-tolerated in ALS patients, and led to improvements of expiratory muscle strength and swallowing kinematics [10]. However, no differences were noted for FVC. The breath stacking technique can increase inspired volumes that lead to greater peak cough flow, allowing for improvements in mucus clearance and reduction in atelectasis (6). We believe that the increase of inspired volume may increase the efficacy of expiratory muscle training in the ALS population.

The American Academy of Neurology, Practice Parameters has recommended that breath stacking technique as a respiratory aid in patients with neuromuscular diseases. This trial aims to determine the impact of an inspiratory and expiratory synergist program in ALS patients using the same resuscitation bag with an addiction of a new valve (VUP) that allows for increased lung capacity and the addiction of a submaximal load to expiratory muscles during the expiration phase, which may provide muscular conditioning in ALS patients and contribute to a maintained bulbar function.

## 3 Conclusion

ALS patients have a poor survival and demonstrate high burden of disease when their bulbar and respiratory function decline. This randomized trial will assess the effects of home-based program in pulmonary and bulbar function. If beneficial, the intervention is designed in way to enable easy translation into practical guidelines for this population.

## Author statement

Alessandra Dorça: Conceptualization, Methodology, Livia A. Alcântara: Investigation, Resources, Denise Sisterolli Diniz: Writing - original draft, Max Sarmet: Investigation, Methodology,Sérgio Ricardo Menezes Mateus: Writing - original draft, Luis Vicente Franco Oliveira: Writing - original draft, Hamilton Franco: Investigation, Resources, Vinicius Maldaner: Conceptualization, Methodology, Writing - original draft

## Funding

No funding or sponsorship was received for this study or publication of this article.

## Declaration of competing interest

All authors declare that have no conflict of interest.
